# Genome Sequence of *Brucella abortus* Vaccine Strain S19 Compared to Virulent Strains Yields Candidate Virulence Genes

**DOI:** 10.1371/journal.pone.0002193

**Published:** 2008-05-14

**Authors:** Oswald R. Crasta, Otto Folkerts, Zhangjun Fei, Shrinivasrao P. Mane, Clive Evans, Susan Martino-Catt, Betsy Bricker, GongXin Yu, Lei Du, Bruno W. Sobral

**Affiliations:** 1 Virginia Bioinformatics Institute, Virginia Tech, Blacksburg, Virginia, United States of America; 2 National Animal Disease Center, Ames, Iowa, United States of America; 3 454 Life Sciences, Branford, Connecticut, United States of America; Ecole Normale Supérieure de Lyon, France

## Abstract

The *Brucella abortus* strain S19, a spontaneously attenuated strain, has been used as a vaccine strain in vaccination of cattle against brucellosis for six decades. Despite many studies, the physiological and molecular mechanisms causing the attenuation are not known. We have applied pyrosequencing technology together with conventional sequencing to rapidly and comprehensively determine the complete genome sequence of the attenuated *Brucella abortus* vaccine strain S19. The main goal of this study is to identify candidate virulence genes by systematic comparative analysis of the attenuated strain with the published genome sequences of two virulent and closely related strains of *B. abortus*, 9–941 and 2308. The two S19 chromosomes are 2,122,487 and 1,161,449 bp in length. A total of 3062 genes were identified and annotated. Pairwise and reciprocal genome comparisons resulted in a total of 263 genes that were non-identical between the S19 genome and any of the two virulent strains. Amongst these, 45 genes were consistently different between the attenuated strain and the two virulent strains but were identical amongst the virulent strains, which included only two of the 236 genes that have been implicated as virulence factors in literature. The functional analyses of the differences have revealed a total of 24 genes that may be associated with the loss of virulence in S19. Of particular relevance are four genes with more than 60bp consistent difference in S19 compared to both the virulent strains, which, in the virulent strains, encode an outer membrane protein and three proteins involved in erythritol uptake or metabolism.

## Introduction

*Brucella* spp. are gram-negative, facultative, intracellular coccobacilli that may cause brucellosis in humans and livestock. The main economic impact of infection in animals is reproductive failure [1], whereas in humans it is undulant fever and if untreated, a debilitating chronic disease [2]. *Brucella* are also identified as potential agricultural, civilian, and military bioterrorism (category B) agents. They are particularly hard to treat as they infect and replicate within host macrophages. Brucellosis in livestock animals is controlled by vaccination [3]. Human brucellosis is treatable with antibiotics, though the course of antibiotic treatment must be prolonged due to the intracellular nature of *Brucella*.

*B. abortus* “strain 19” or S19 (here after, S19) is a spontaneously attenuated strain discovered by Dr. John Buck in 1923 [4], [5]. The underlying molecular or physiological mechanisms causing the loss of virulence are not well understood. Live, attenuated strain S19 had been used worldwide since the early 1930s as an effective vaccine to prevent brucellosis in cattle, until it was replaced by strain RB51 during the 1990s. S19 maintains its smooth appearance derived from the presence of the extracellular lipopolysaccharide (LPS) while the other vaccine strain, RB51, with rough characteristics devoid of O-chain, does not elicit antibodies against the O-side polysaccharide [6], [7]. Further attenuation or optimization of S19 will be necessary to develop a human vaccine strain, which could be approved through the “Animal Rule” regulatory mechanism [8]. Such modification could result from the expression of additional vaccine candidate proteins to enhance vaccine efficacy [9], or from the inactivation of genes encoding additional virulence factors to reduce residual virulence found in humans.

There are four papers describing the genomes from the following different strains/species of *Brucella*: *B. melitensis* , *B. suis*, *B abortus* strain 2308 (here after 2308), and *B. abortus* strain 9–941 (here after 9–941) [10]–[13]; in addition, two more genome sequences of *Brucella sp.* have been sequenced and have been made available by the Pathosystems Resource Integration Center (PATRIC, http://patric.vbi.vt.edu/). All sequenced strains to date are virulent. Therefore, we set out to sequence the first genome of an attenuated, live vaccine strain, with the main objective of identifying the genes associated with the virulence or lack thereof, through comparison of the newly sequenced genome with that of the virulent counterparts.

We used pyrosequencing (454 Life Sciences Corporation) [14] together with conventional sequencing to determine the complete genome sequence of S19. Here we have described the newly sequenced genome and identification of unique genes by its comparison to genomes of virulent strains of *B. abortus*, 2308 and 9–941 [10], [12]. Comparative genomic analysis identified a number of candidate genes that can be mutated with the aim of further attenuating wild-type or other vaccine strains.

## Results and Discussion

The combination of pyrosequencing and Sanger sequencing allowed for the rapid (one day of sequencing) and comprehensive (more than 99.5% of the genome) closure and assembly of the genome of S19. The average length of the sequence reads was 110 bp. Using the Roche GS-FLX™ we were able to improve read lengths to an average of 230 bp (data not shown).

### Genome Sequence Properties

The 3.2 Mb S19 genome is comprised of two circular chromosomes (Table 1): one 2,122,487 bp long and the other 1,161,449 bp long. The average GC content of the two chromosomes is 57%. Not surprisingly, S19's genome showed remarkable similarity in size and structure to those of its virulent relatives, *B. abortus* 9–941 and 2308. The size of the S19 genome is within 5 kb of 9–941 (3.283Mbp) and 2308 (3.278 Mbp) genomes. The S19 genome sequence shows over 99.5% similarity compared to the genomes of 9–941 and 2308.

**Table 1:** Genome properties of the newly sequenced genome of *B. abortus* strain S19 in comparison with the known genome sequence of two virulent strains.

	*B. abortus* S19		*B. abortus* 9–941(a)		*B. abortus* 2308(a)	
Feature/Property	ChrI	ChrII	ChrI	ChrII	ChrI	ChrII
ORFs	2,005	1,057	2,030	1,055	2,000	1,182
tRNA	41	14	41	14	44	15
rRNA	6	3	6	3	6	3
Size	2,122,487	1,161,449	2,124,241	1,162,204	2,121,359	1,156,948
GC (%)	57.2	57.3	57.2	57.3	57.2	57.3
Average gene length	296.8	314.2	281.9	300.0	284.0	301.4
Coding (%)	28.2	28.9	26.9	27.2	26.8	26.9
Conserved hypothetical	0	0	11	8	0	0
Hypothetical proteins	408	163	708	296	526	217

a. The source of the data are Halling et al., 2005 [12]; Chain et al., 2006 [10] or PATRIC website (http://patric.vbi.vt.edu/)

**Figure 1:**
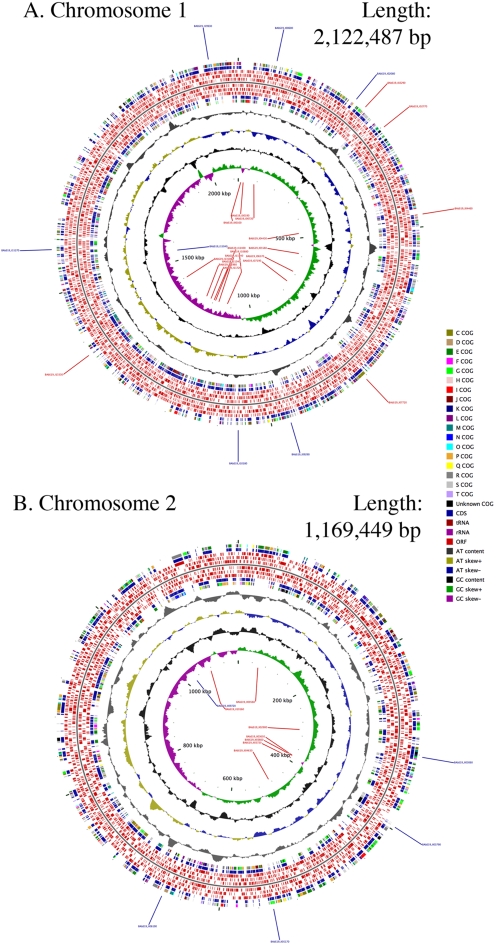
Complete DNA sequence of *Brucella abortus* strain S19. The concentric circles show, reading outwards: GC skew, GC content, AT skew, AT content, COG classification of proteins, CDS on reverse strand, ORFs on three frames in reverse strand, ORFs on three frames in forward strand, CDS on forward strand and COG classification of proteins on forward strand. The genes that differ from both 2308 and 9–941 strains are labeled.

**Figure 2:**
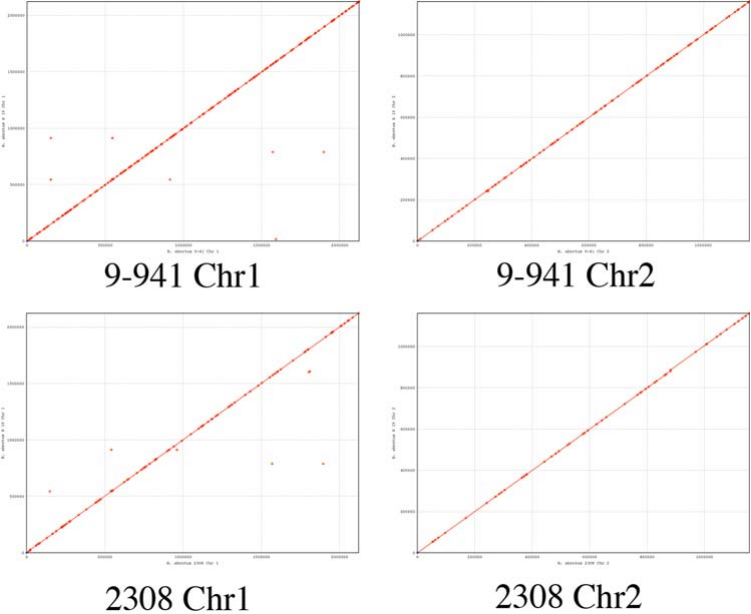
Comparative genomic analysis of the *Brucella abortus* strain S19 with the virulent strains 9–941 and 2308 using the MUMer program.

The S19 chromosomes and their comparison to the 9–941 and 2308 chromosomes are shown in Figure 1. The online version of the Figure 1 is interactive (and is available at http://patric.vbi.vt.edu/). A total of 2,047 and 1,086 open reading frames (ORFs) were identified on the first and second chromosomes, respectively. More than 96% of these ORFs are identical to the ORFs of 9–941 and 2308. Functional assignment of the ORFs was carried out by BLASTP searches against four *Brucella* genomes. S19 exhibits very high genome-wide collinearity with 9–941 and 2308 in Chromosome 1. Chromosome 2 has perfect collinearity with both 9–941 and 2308 (Figure 2.). Of the 3,062 predicted ORFs, more than 79% had BLASTP hits to the cluster of orthologous groups (COG) database with an e-value less than 1e–4. A total of 571 ORFs (18%) are hypothetical proteins (Table 2 and Supplemental Table S1).

**Table 2:** COG-based functional categories of *B. abortus* S19 coding sequences.

	No. of ORFs		
Functional Category (NCBI COGs)	ChrI	ChrII	Total
INFORMATION STORAGE AND PROCESSING			
Translation, ribosomal structure and biogenesis	139	25	164
RNA processing and modification	0	0	0
Transcription	79	50	129
Replication, recombination and repair	86	21	107
Chromatin structure and dynamics	0	0	0
CELLULAR PROCESSES AND SIGNALING			
Cell cycle control, cell division, chromosome partitioning	19	8	27
Nuclear structure	0	0	0
Defense mechanisms	18	17	35
Signal transduction mechanisms	38	16	54
Cell wall/membrane/envelope biogenesis	114	28	142
Cell motility	3	17	20
Cytoskeleton	0	0	0
Extracellular structures	0	0	0
Intracellular trafficking, secretion, and vesicular transport	25	10	35
Posttranslational modification, protein turnover, chaperones	100	31	131
METABOLISM			
Energy production and conversion	97	61	158
Carbohydrate transport and metabolism	67	83	150
Amino acid transport and metabolism	158	106	264
Nucleotide transport and metabolism	47	16	63
Coenzyme transport and metabolism	100	20	120
Lipid transport and metabolism	59	20	79
Inorganic ion transport and metabolism	68	65	133
Secondary metabolites biosynthesis, transport and catabolism	14	16	30
POORLY CHARACTERIZED			
General function prediction only	188	81	269
Function unknown	203	66	269
No similarity to COGs with an e-value lower than 1e–4	344	276	620

Genome wide comparisons were between S19 and the virulent 9–941 genomes to identify all the single nucleotide polymorphisms (SNPs) between the two genomes. A more stringent criteria was used to identify SNPs using all the sequenced reads, then to identify the differences in ORFs (sections below). A total of 201 SNPs were identified in S19 when compared to 9–941, and are listed in Table 3. The exact position and alignment of all the SNPs at the nucleotide level and protein level (if the SNP is within an ORF) is given in the Supplemental Table S2. Forty-four of the 201 SNPs (22%) were located in intergenic regions. Forty-nine SNPs were synonymous substitutions, encoding the same amino acid (aa). Sixty-five SNPs were conservative, non-synonymous substitutions, encoding a different aa with similar properties. Radical non-synonymous substitutions were found in 36 of the SNPs, resulting in the incorporation of an aa with a net change in charge or polarity. These SNPs between S19 and 9–41 were also compared for consistency by comparing the S19 genome to 2308 genome. A total of 39 single nucleotide differences in ORFs that were consistently different between S19 and its two virulent counterparts and their relevance to virulence are described in the section below.

**Table 3:** Single nucleotide polymorphisms detected in *B. abortus* strain S19 as compared to the strain 9–941.

	S19 nt					
9–941 nt	-	a	c	g	t	Total
Chr I						
-		0	1	0	0	1
a	1		2	27	2	32
c	4	9		9	16	38
g	2	21	2		10	35
t	2	0	20	8		30
ChrII						
-						0
a	0		1	14	0	15
c	2	3		1	9	15
g	0	15	3		3	21
t	0	2	11		1	14
Total	11	50	40	59	41	201

### Identification of Virulence Associated Differences between the Attenuated and Virulent Strains

The main focus of the work was to identify all the ORFs that are different in S19 when compared to its virulent relatives, 9–941 and 2308, which provide a complete basis for the attenuation of S19. Pairwise and reciprocal comparisons were made between S19 and the published genome sequences of 9–941 and 2308, to identify genes that are 100% identical and consequently, those non-identical ORFs with any differences (Table 4). In each pairwise comparison, predicted genes from one genome were aligned to the whole genome sequence of the second strain and *vice versa*, to allow for differences in gene annotation among the genomes. In each of the genes-to-genome pairwise comparisons, more than 95% of the genes from strain-1 were identical to a corresponding sequence from the whole genome of strain-2. The bulk of the rest of the genes (an average of 4%) had only one nucleotide (nt) difference, while less than 1% of the genes showed differences of more than 1 nucleotide. The number of genes that were non-identical was greater in the comparison between S19 and 9–941 than in the comparison between S19 and 2308. The results of the pairwise and reciprocal comparisons between S19, 9–941 and 2308 are given in the Supplemental Table S3.

**Table 4:** Reciprocal gene to genome pairwise comparison between *B.abortus* strains S19, 9–941, and 2308.

		Number of nucleotide-differences *							
Comparison from:	Comparison to:	Identical	Non-Identical						
Strain-1	Strain-2	0	1	2	3	4	> = 5	total	Total ORFs in strain-1
9–941	S19	2892 (94)	167 (5.4)	4 (0.1)	0 (0)	0 (0)	12 (0.4)	183 (6)	3075 (100)
S19	9–941	2810 (93.8)	170 (5.7)	5 (0.3)	1 (.03)	0 (0)	10 (0.3)	186 (6.2)	2996 (100)
2308	S19	2934 (97)	78 (2.6)	3 (0.1)	0 (0)	0 (0)	8 (0.3)	89 (3)	3023 (100)
S19	2308	2895 (96.6)	84 (2.8)	3 (0.1)	0 (0)	0 (0)	14 (0.5)	101 (3.4)	2996 (100)
Average		2882.75 (95.38)	124.75 (4.1)	3.75 (0.1)	0.25 (0)	0 (0)	11 (0.4)	139.75 (4.6)	3022.5 (100)

* the numbers in parentheses are percentages of the total number of predicted genes in strain-1

The pairwise and reciprocal genes-to-genome comparisons allowed us to account for the genes that were predicted in one genome but not predicted in the other. A total of 260 and 214 ORFs identified in 9–941 and 2308, respectively, were not predicted in S19. More than 95% of these sequences were identical to S19 and more than 91% of these were annotated as “hypothetical”. Similarly, of the ORFs identified in S19 a total of 321 and 311 were not predicted in 2308 and 9–941, respectively, and more than 90% of these S19 sequences were identical to those in the 2308 and 9–941 genomes. As expected, the differences in number of ORFs between the three strains were much smaller when the same methods were applied for ORF prediction (data not shown). The differences in the gene annotations are corrected through the curation efforts by the PATRIC project and the new annotations are available at http://patric.vbi.vt.edu/.

Comprehensive pairwise and reciprocal genes-to-genome comparisons of all the predicted ORFs in the three genomes of S19, 2308 and 9–941 revealed only 263 ORFs that were non-identical (<100% homology) between S19 and any of the two virulent genomes, 9–941 and 2308. The data are summarized in Table 5 and the details of the genes and their differences are given in the Supplemental Table S4. Of the 263 ORFs identified as non-identical between S19 and any of the two virulent strains (Table 5), 70 ORFs showed nucleotide changes but not aa changes, therefore they were not further pursued from the perspective of explaining the differences in virulence. A total of 148 ORFs showed differences between S19 and only one of the two virulent strains, while there was no difference compared to the other. This included some of the ORFs that have been implicated in virulence (.e.g., AroA, 3-phosphoshikimate 1-carboxyvinyltransferase), but are not discussed because of their indifference in one of the two virulent strains. The remaining 45 ORFs showed consistent sequence deviation in S19 compared to both the virulent strains, while both virulent strains maintained identical sequences. These 45 ORFs that were consistently different between the attenuated S19 and the two virulent strains were evaluated to identify candidate virulence associated ORFs. The clusters of orthologous groups of proteins ( COG) functional classification of the 45 ORFS, with consistent differences between S19 and both the virulent strains (OCDs), and all of the 263 non-identical differences, is shown in Table 6. While the non-identical differences were distributed in a total of 19 COG classes, the OCDs were clustered in 11 COG classes (excluding no hits and unknown).

**Table 5:** Number of genes identified to be different between attenuated strain S19 genome and the virulent strains 9–941 and 2308.

			Consistency- Different between:			
Category	Priority*	Nucleotide difference	S19 and 9–941	S19 and 2308	9–941 and 2308	No. of genes
Consistent Differences between S19 and the two virulent strains	0	>10 nt	Yes	Yes	No	4
	1	2–10 nt	Yes	Yes	No	2
	2–4	1 nt	Yes	Yes	No	27
	5**	1nt	Yes	Yes	No	12
Other Differences	6	> = 1nt	-	No	-	108
	7	> = 1nt	No	-	-	32
	8	> = 1nt	Yes	Yes	Yes	9
	9	> = 1nt	no aa change	no aa change	-	69
Total						263

* See the Supplemental Table S4 for list of genes and the summary of the differences

** Sequencing variation (variablity in the reads) and transposases

**Table 6:** COG-based functional categories of ORFs identified as differences between S19 and the virulent *B. abortus* strains 9–941, and 2308.

COG-Category		All Differences	Virulence Associated Differences
I	Lipid transport and metabolism	11	5
K	Transcription	14	4
E	Amino acid transport and metabolism	30	3
L	Replication, recombination and repair	9	1
O	Posttranslational modification, protein turnover, chaperones	9	3
P	Inorganic ion transport and metabolism	11	2
C	Energy production and conversion	15	2
G	Carbohydrate transport and metabolism	15	2
R	General function prediction only	20	2
F	Nucleotide transport and metabolism	2	1
J	Translation, ribosomal structure and biogenesis	15	1
D	Cell cycle control, cell division, chromosome partitioning	5	0
H	Coenzyme transport and metabolism	7	0
M	Cell wall/membrane/envelope biogenesis	9	0
N	Cell motility	3	0
Q	Secondary metabolites biosynthesis, transport and catabolism	2	0
T	Signal transduction mechanisms	9	0
U	Intracellular trafficking, secretion, and vesicular transport	1	0
V	Defense mechanisms	7	0
S	Function unknown	20	3
-	No COG Hits	59	13
Total		263	45

The details on the differences between S19 and the two virulent strains of all the 45 OCDs are shown in Table 7. Four of the 45 OCDs (ORFs BruAb1_0072, BruAb2_0365, BruAb2_0366, and BruAb2_0372 in 9–941) showed more than 60 bp differences between the attenuated and the virulent strains and were considered as “Major Virulence Associated Differences” (“priority 0” in Table 7). The remaining 41 OCDs had only less than 10 bp difference and hence were considered as “Minor OCDs”. All the major virulence associated differences and some of the OCDs with possible association to virulence are described in the sections below. Further follow up experiments are being designed to test the functions of some of the virulence associated ORFs by mutation studies of S19 and the virulent strains and their responses during infection, and hence they are not part of this manuscript.

**Table 7:** List of ORFs with consistent differences (OCDs) between the attenuated strain S19 and the two virulent strains, 2308 and 9–941, but not different within the virulent strains.

slno	priority	Locus*	9–941 vs. S19	2308 vs. S19	aa change**	Annotation***
1	0	BruAb1_0072	790 Θ	1695 Θ		S_outer membrane protein
2	0	BruAb2_0365	247 Θ	247 Θ		K_erythritol transcriptional regulator
3	0	BruAb2_0366	423 Θ	423 Θ		-_EryC, D-erythrulose-1-phosphate dehydrogenase
4	0	BruAb2_0372	68 Θ	68 Θ		G_ribose ABC transporter, permease protein
5	1	BruAb1_0019	7 Θ	7 Θ	FS	I_carboxyl transferase family protein
6	1	BruAb1_0443	2 Ψ	2 Ψ	FS	I_enoyl-(acyl carrier protein) reductase
7	2	BAB1_0967	1 Ψ	1 Ψ	PS	I_Membrane protein involved in aromatic hydrocarbon degradation
8	2	BruAb1_0229	1 Θ	1 Θ	FS	K_transcriptional regulator, IclR family
9	2	BruAb2_0379	1 Δ	1 Δ	FS	O_hypothetical epimerase/dehydratase family protein
10	2	BruAb1_1172	1 Θ	1 Θ	FS	C_glycerophosphoryl diester phosphodiesterase family protein
11	2	BruAb2_1016	1 Δ	1 Δ	FS	-_aldehyde dehydrogenase family protein
12	2	BAbS19_I18830	1 Ψ 3 Δ	1 Ψ	FS	-_intimin/invasin family protein
13	3	BruAb1_1504	1 Ψ	1 Ψ	D->N(yes)	L_excinuclease ABC subunit B
14	3	BruAb1_0772	1 Ψ	1 Ψ	Q->R(yes)	O_arginyl-tRNA-protein transferase
15	3	BruAb1_1114	1 Ψ	1 Ψ	E->Q(yes)	O_ATP-dependent protease ATP-binding subunit
16	3	BruAb1_0208	1 Ψ	1 Ψ	E->Q(yes)	P_ABC transporter, periplasmic substrate-binding protein
17	3	BruAb2_0517	1 Ψ	1 Ψ	R->H(yes)	G_IolC myo-catabolism protein
18	3	BruAb1_1068	1 Ψ	1 Ψ	R->H(yes)	R_hypothetical protein
19	3	BAbS19_I18870	1 Ψ	1 Ψ	E->G(yes)	R_outer membrane autotransporter
20	3	BruAb1_1140	1 Ψ	1 Ψ	S->R(yes)	F_CTP synthetase
21	3	BruAb1_0060	1 Ψ	1 Ψ	R->L(yes)	-_transcriptional regulator, LysR family
22	3	BruAb1_0657	1 Ψ	1 Ψ	D->G(yes)	-_Omp2b, porin
23	3	BruAb1_0719	1 Ψ	1 Ψ	N->D(yes)	-_short chain dehydrogenase
24	3	BruAb2_0290	1 Ψ	1 Ψ	S->R(yes)	-_hypothetical protein
25	4	BAbS19_II07300	2 Ψ	1 Ψ	V->I(no)	I_ACETYL-COENZYME A SYNTHETASE
26	4	BruAb2_1104	1 Ψ	1 Ψ	S->P(no)	S_hypothetical protein
27	4	BruAb1_0010	1 Ψ	1 Ψ	A->V(no)	E_ABC transporter, periplasmic substrate-binding protein, hypothetical
28	4	BruAb1_1018	1 Ψ	1 Ψ	A->T(no)	E_Dhs, phospho-2-dehydro-3-deoxyheptonate aldolase, class II
29	4	BruAb1_1993	1 Ψ	1 Ψ	V->M(no)	P_CadA-1, cadmium-translocating P-type ATPase
30	4	BruAb1_1134	1 Ψ	1 Ψ	N->S(no)	C_dihydrolipoamide acetyltransferase
31	4	BruAb1_0277	1 Θ	1 Ψ	T->P(no)	J_translation initiation factor IF-1
32	4	BruAb1_1196	1 Ψ	1 Ψ	A->S(no)	-_hypothetical protein
33	4	BruAb2_0463	1 Ψ	1 Ψ	I->F(no)	-_hypothetical protein
34	5	BruAb1_1527	1 Θ	1 Θ	FS	I_maoC-related protein
35	5	BruAb2_0306	1 Ψ	1 Ψ	A->P(no)	K_HutC, histidine utilization repressor
36	5	BruAb2_0619	1 Ψ	1 Ψ	R->C(yes)	K_exoribonuclease, VacB/RNase II family
37	5	BruAb1_1333	1 Δ	1 Δ	FS	S_dedA family protein
38	5	BruAb2_0056	1 Θ	1 Θ	FS	E_amino acid permease family protein
39	5	BruAb2_0972	1 Δ	1 Δ	FS	-_hypothetical membrane protein
40	5	BruAb1_1141	1 Δ	1 Δ	FS	-_hypothetical protein
41	5	BAbS19_I08740	1 Ψ	1 Ψ	V->E(yes)	-_hypothetical protein
42	5	BruAb1_0132	1 Ψ	1 Ψ		S_IS711, transposase orfB
43	5	BruAb1_0929	1 Ψ	1 Ψ		L_IS711, transposase orfB
44	5	BruAb1_0556	1 Ψ	1 Ψ		-_transposase orfA
45	5	BruAb1_1835	1 Δ	1 Θ		-_IS2020 transposase

* Representative locus from 9–941, S19 or 2308 genomes. The mapping for all three genomes is given in Supplemental Table S4

** Ψ = bp Difference, Δ  = bp Deletion, Θ = bp insertion,FS:Frameshift, PS:prematurestop, other letter indicate aa, the word “yes” or “no” in parenthesis indicates if the change in aa caused change in net charge

*** The first character indicates the COG category (see Table 6)

### Major Virulence Associated Differences

**Figure 3:**
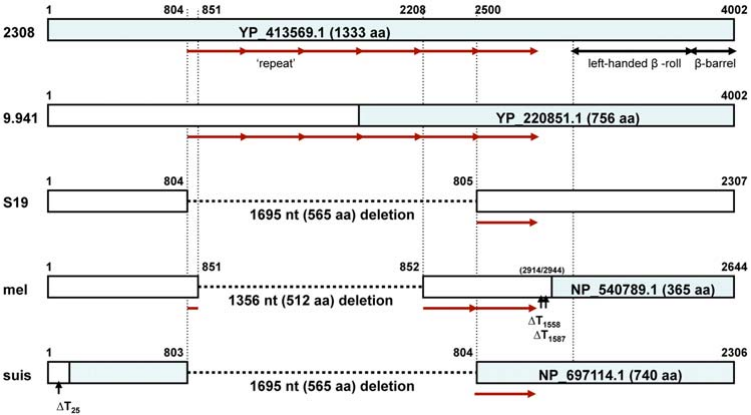
Diagramatic representation of the alignment of the outer membrane protein loci (BAB1_0069) across sequenced *Brucella* genomes.

Rearrangement in an outer membrane protein: The most striking and consistent difference between S19 and the two virulent strains was in the region of the ORF, BAB1_0069 of 2308, encoding a putative 1,333 aa outer membrane protein. Compared to both 2308 and 9–941, this locus in S19 suffers a 1,695 nt. deletion, corresponding to nucleotides 805–2499 of BAB1_0069. The deletion removes amino acids 269–833 and therefore the predicted ORF in S19 is only 768 aa long (Figure 3).

A structural similarity search against the Protein Data Bank revealed two significant hits to *Yersinia enterocolitica* YadA and the *Haemophilus influenzae* Hia adhesins, respectively. Both YadA and Hia are important virulence factors involved in adhesion, invasion, and serum resistance. The structural similarity between YadA and BAB1_0069 resides in amino acids 62–265 of YadA and 1064–1251 of BAB1_0069. This region in YadA was recently shown to consist of a globular head region consisting of nine-coiled left-handed parallel β-rolls (LPBR) [15]. Phagocytic uptake by host macrophages is clearly essential for a robust immune response, and evasion of this uptake is a protective mechanism for extra-cellular pathogens such as the *Yersiniae*. *Brucella* on the other hand are intracellular pathogens and infect initially and mainly macrophages, and therefore depend on an efficient mechanism for adhesion and cellular uptake.

Although the 1,695 nt deletion in the outer membrane protein is consistent between S19 and both the virulent strains, further studies are needed to associate this protein to the lack of virulence in S19, as further examination of the same ORF in other virulent species of Brucella reveals the presence of similar deletions (Figure 3) in *B. melitensis* and *B. suis*. The *B. melitensis* locus contains 3 deletions. The first deletion results in a 512 aa deletion. The locus also has two single nucleotide deletions, causing a frame shift, such that the ortholog in *B. melitensis* contains only the terminal 365 aa (NP_540789.1). The *B. suis* genome has two deletions, resulting in an ORF predicted to encode 740 aa. The deletions in S19 and *B. suis* are identical in length and position and further examination of the nucleotide and protein sequences and alignments revealed the presence of a tandem repeat sequence of 339 nucleotides (113 aa). Six copies of the repeated sequence occur in the full-size ORFs of strains 2308 and 9–941 (Figure 3). The variant ORFs could have arisen by deleting portions of the coding regions/proteins through ‘recombination’ between the 1^st^ and 6^th^ repeat sequence in strain S19 and *B. suis*, and the 1^st^ and 5^th^ repeat in *B. melitensis*. Although the presence of similar deletions in other virulent species eliminates the possibility of association of the deletion to lack of general virulence, it is possible that the deletion may be associated with species specific (*B. abortus*) host preference, which needs to be tested in mutation experiments.

**Figure 4:**
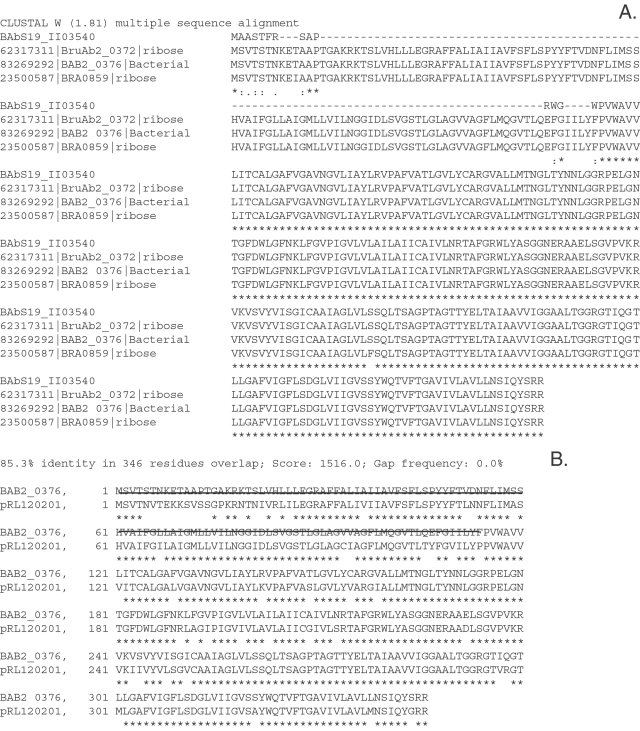
Protein alignment of the ribose transporter protein, S19 BAbS19_II03540; A. with the homologs from strain 9–941 (BruAb2_0372), 2308 (BAB2_0376) and B. suis (BRA0859); B. Protein alignment of the ribose transporter protein from strain 2308 (BAB2_0376) with the putative *Rhizobium leguminosarum* eryF protein. The sequence deleted in S19 BAbS19_II03540 is indicated by the strike-through mark-up of the 2308 sequence.

Rearrangements in the erythritol catabolic operon (eryC, eryD) and related transporter (eryF): The second largest gene rearrangement in S19 compared to both the virulent strains, 2308 and 9–941, occurs in the erythritol (ery) operon. The erythritol operon contains 4 ORFs for eryA, eryB, eryC and eryD respectively. Compared to the virulent strains, S19 has a 703 nucleotide deletion which interrupts both the coding regions of eryC (BAB2_0370) and eryD (BAB2_0369). The deletion affects the C terminal part of eryC and the N-terminal part of eryD proteins from *B. abortus* strains 2308 (BAB2_0369), 9–941 (BruAb2_0365) and *B. suis* (BRA0867). Figure 4A shows the alignment of the predicted protein with the C-terminal part of eryD. The deletion in eryC and eryD ORFs of S19 has been previously shown [16], [17]. The importance of this deletion in the attenuation of S19 has also been studied using Tn*5* insertions and complementation analysis, revealing that it is not sufficient or required for virulence in a mouse model [18].

Eryrthritol metabolism by *Brucella* has been identified as a trait associated with the capability of the pathogen to cause abortions in livestock. The preferential growth of *Brucella* in the foetal tissues of cattle, sheep, goats and pigs was also shown to be due to the high concentration of erythritol [19]. Although *Brucella* infects and cause brucellosis in other organisms such as human, rat, rabbit and guinea pig, overwhelming infection of the placental and foetal tissues is not observed, which is also associated with low concentrations of erythritol [19]. According to Garcia-Lobo and Sangari [20], the cultures of “strain 19” provided by the USDA before 1956 showed differences in growth in the presence of erythritol and in erythritol oxidation rates. During that time, erythritol sensitive cultures were selected and used to substitute the previous batches of vaccine, which was then renamed “US19” or just “S19”. Jay F. Sperry and Donald C. Robertson [21] elucidated the pathway of erythritol catabolism in *Brucella* using radiolabelling experiments. Later, Tn*5* mutagenesis of virulent 2308 revealed four *ery* genes (*ery*ABCD) proposed to exist as an operon [16]. The *ery* operon in S19 was also analyzed and shown to contain a deletion of 702 bp affecting two genes, *ery*C and *ery*D [17].

In a murine model, when attenuated S19 and virulent 2308 strains were compared to genetically engineered strains, including: (1) a knock-out mutant of 2308 (Δ*ery*CD), (2) a naturally reverted S19 strain (ery resistant), and (3) S19 strain transformed with the wild type *ery* operon, there was no direct correlation between erythritol metabolism and *in vivo* colonization [18]. However, the experiments that have been performed in mice do not address the question of whether or not an eryCD mutation would attenuate *B. abortus* 2308 in a pregnant ruminant as the *B. abortus* dhbC mutant is shown to be extremely attenuated in pregnant cattle [22]. These results suggest that S19 has lost some additional and essential, yet unknown, mechanism of virulence in mice.

S19 also contains a 68 nucleotide deletion in the ORF corresponding to BAB2_0376 of *B. abortus* 2308, which results in a 114 aa N terminal truncation (Figure 4 AB). BAB2_0376 encodes a putative inner-membrane translocator sodium dicarboxylate symporter, which is highly conserved among many gram negative bacteria, proteobacteria, enterics and gram positive bacteria. Christopher K. Yost et al. [23] recently characterized a region of the *Rhizobium leguminosarum* 3841 responsible for erythritol uptake and utilization, which is highly conserved within the corresponding genome region of *Brucella* spp. In *R. leguminosarum* 3841 the eryABCD erythritol catabolic operon is flanked by a putative operon containing a hypothetical protein, a putative nucleotide binding protein (EryE), permease (EryF) and a periplasmic binding protein (EryG). Transposon mutation of *eryF* abolished erythritol uptake. Because BAB2_0376 has 85% sequence similarity with the predicted eryF ORF (pRL120201) and the conservation of gene order with the eryEFG operon, it is very likely that BAB2-0376 also encodes a putative erythritol transporter. Hence, we hypothesize that mutation of the gene in S19, in addition to the large deletion in the eryABCD operon, further contributes to the inability of S19 to metabolize erythritol. Further experiments are designed to test the combined impact of the eryCDF on the attenuation of the virulent strains.

### Minor Virulence Associated Differences

As shown in Table 7, a total of 41 ORFs were identified as minor OCDs, which showed a consistent difference of less than 10bp between the attenuated S19 strain and both the virulent strains, 9–941 and 2308. These ORFs were grouped into 11 classes based on the COG classification. Only two minor OCDs, carboxyl transferase (BruAb1_0019) and Enoyl-acyl-carrier proteins and (BruAb1_0443) showed more than 1 bp difference (“priority 1” in Table 7) resulting in frameshifts, while the remaining 39 minor OCDs showed only single nucleotide differences (“priority 2 to 4” in Table 7). Among the 39 minor OCDs that showed single nt differences, 12 ORFs showed either variation among reads within S19 or were transposases (“priority 5 to 4” in Table 7) and hence were omitted from discussion. Six OCDs had frameshifts between S19 and the two virulent strains (“priority 2” in Table 7), which included one protein involved in Lipid transport and metabolism (BAB1_0967), an IclR family transcriptional regulator (BruAb1_0229), aUDP-glucose 4-epimerase (BruAb2_0379), a Glycerophosphodiester phosphodiesterase domain-containing protein (BruAb1_1172), an aldehyde dehydrogenase family protein (BruAb2_1016), and an intimin/invasin family protein (BAbS19_I18830). Some of the 12 minor OCDs (“priority 3” in Table 7) that resulted in the net charge change included excinuclease ABC subunit B (BruAb1_1504), ABC transporter, periplasmic substrate-binding protein (BruAb1_0208), IolC myo-catabolism protein (BruAb2_0517), outer membrane autotransporter (BAbS19_I18870), CTP synthetase (BruAb1_1140), Omp2b, porin (BruAb1_0657), and three hypothetical proteins. We also identified 9 OCDs that showed amino acid changes without change in the net charge (“priority 4” in Table 7).

Some of the minor OCDs and their relevance to attenuation (besides the four major differences) are described below. Although we have discussed the association of some of the OCDs to the virulence (in the virulent strains) or lack there of (in S19), further mutation experiments are needed and are underway to verify their functional relevance. As mentioned before, the main focus of this project is to catalog all the consistent differences between the attenuated strain S19 and the two virulent strains (Table 7).

We identified a total of four ORFs under the classification of lipid transport and metabolism. They are: carboxyl transferase family protein (BruAb1_0019), enoyl-[acyl-carrier-protein] reductase [NADH] (BruAb1_0443), membrane protein involved in aromatic hydrocarbon degradation (BAB1_0967), and acetyl-coenzyme a synthetase (BAbS19_II07300). The differences in the first four OCDs caused frameshift or premature stop codons in the S19 ORF, while the difference in the last caused change in aa (V to I) without any change in the net charge. The impact of the difference in carboxyl transferase family protein needs a follow up to assess its impact on the virulence, as *B. abortus* encodes two additional carboxyl transferases, BAB1_1210 (510 aa) and BAB1_2109 (AccD, 301 aa). It is not clear if the mutation in BAB1_0019 may be compensated for by the presence of two additional ACCase beta subunit genes. *Mycobacterium tuberculosis* encodes six AccDase proteins, and at least three of these have a putative role in virulence [24]–[27]. The locus encoding enoyl-[acyl-carrier-protein] reductase [NADH] (BruAb1_0443) showed 2 bp difference between S19 and the two virulent strain, while the other two ORFs (BAB1_0967, and BAbS19_II07300) showed single bp difference.

The locus encoding transcriptional regulator, IclR family (BruAb1_0229) contains a one base deletion in S19, causing a frameshift in the C-terminus and premature termination of the ORF, changing the C-terminal 28 aa of BruAb1_0229. BruAb1_0229 encodes a 264 aa protein with homology to the IclR family of regulatory proteins [28]. The C-terminal half of the protein contains the Helix-turn-helix DNA binding motif [29]. The change of the C-terminal 28 aa in the S19 protein removes or changes Helix 9 of the TM-IclR structure.

### Comparison of the differences between S19 and the virulent strains to the Brucella virulence factors

As mentioned before, the main focus of this project was to identify candidate ORFs that are associated with the virulence (in 9–941 and 2308) or lack there of (in S19). A comprehensive analysis of the genomes revealed 263 non-identical differences. As described above, we have identified 45 ORFs that were consistently different between S19 and the two virulent strains. As an independent evaluation, we also compared all the 263 non-identical differences (as listed in the Supplemental Table S4) to the virulence factors that are described in literature as experimentally characterized in *Brucella*, which is described below.

Large-scale screens and testing in model systems have been performed in *Brucella* to identify virulence factors associated with the pathogenesis and virulence. Rose-May Delrue et al., [30] studied the literature and identified a total of 192 virulence factors that have been characterized using 184 attenuated mutants, as well as an additional 44 genes, which have been characterized for virulence in *Brucella* by different groups [31]–[41]. The sequences of all these 236 genes were compared to the 263 ORFs that we have identified as non-identical differences between S19 and any of the two virulent strains of *B. abortus*. The comparison yielded a total of 26 ORFs that were non-identical between S19 and any of the two virulent strains (<100% homology) as shown in Table 8. However, out of the 22, only three ORFS (BRA0866:BAB2_0370, BRA1168:BAB2_1127, and BR1296:BruAb1_1350) were identified as showing consistent differences between S19 and the two virulent strains (9–941 and 2308). Amongst these only two (BAB2_0370, BAB2_1127) were included as virulence associated ORFS, as the third one did not show difference at the amino acid level. The ORF, BAB2_0370 (*Ery*C), was identified as a major virulence associated difference and is described in detail in the above section. The other ORF, BAB2_1127, which encodes a hypothetical protein associated with UPF0261 protein CTC_01794 (COG5441S) in *B melitensis* (BMEII0128) was screened in a murine infection model through signature-tagged mutagenesis (STM), and was used to identify genes required for the in vivo pathogenesis of *Brucella* [42] with a combined attenuation score of 4 [30]. None of the other ORFs listed in Table 8 were consistently different between S19 and the two virulent strains.

**Table 8:** Comparison of the list experimentally characterized *Brucella* virulence factors to the non-identical differences between S19 and the virulent strains.

slno	Pr.	Virulence Factor*	Gene Symbol	Ref	ATT	*B. abortus* ortholog	9–941 vs. S19	2308 vs. S19	2308 vs. 9–941	Product Description
3	0	BRA0866	eryC	30	−3	BAB2_0370	423 Λ	423 Λ	Ϊ	Uptake hydrogenase large subunit
26	5	BRA1168	hypo	42	4	BAB2_1127	1 Ψ	1 Ψ	Ϊ	UPF0261 protein CTC_01794
69	6	BR0139	unkn	30	10	BruAb2_0179	1 Ψ	Ϊ	1 Ψ	Hypothetical protein
83	6	BRA1012	dppA	30	10	BAB2_0974	1 Ψ	Ϊ	1 Ψ	Heme-binding protein A precursor
90	7	BR1053	cysK	42	10	BAB1_1968	Ϊ	7 Λ	7 Λ 1 Ψ	Cysteine synthase
97	9	BR0188	metH	42	10	BAB1_0188	1 Ψ	Ϊ	1 Ψ	methionine synthase
110	6	BRA1146	fliF	30	3	BAB2_1105	1 Ψ	Ϊ	1 Ψ	flagellar M-ring protein FliF
120	8	BR0181	cysI	42	8	BAB1_0181	1 Ψ	2 Ψ	1 Ψ	Hypothetical protein
128	7	BR0436	dxps	30	1	BAB1_0462	Ϊ	1 Ψ	1 Ψ	1-deoxy-D-xylulose-5-phosphate synthase
131	7	BRA0299	narG	30	3	BAB2_0904	Ϊ	1825 Λ	1 Ψ	nitrate reductase, alpha subunit
135	6	BR0111	ndvB	30	7	BAB1_0108	1 Ψ	Ϊ	1 Ψ	Hypothetical protein
136	6	BR0537	pmm	30	10	BAB1_0560	1 Ψ	Ϊ	2 Ψ	Phosphomannomutase
139	6	BRA0806	galcD	30	10	BAB2_0431	1 Ψ	Ϊ	1 Ψ	Hypothetical protein
143	9	BR0866	rbsK	30	9	BAB2_0004	1 Ψ	Ϊ	1 Ψ	ribokinase
145	9	BRA0936	araG	30	−2	BAB2_0299	Ϊ	1 Ψ	1 Ψ	L-arabinose transport ATP-binding protein araG
154	6	BRA0987	cobW	30	3	BAB2_0246	1 Ψ	Ϊ	425Λ1 Ψ	COBW domain-containing protein 1
191	6	BR0511	wbpL	30	9	BAB1_0535	1 Ψ	Ϊ	1 Ψ	Phospho-N-acetylmuramoyl-pentapeptide-transferase
198	9	BR1671	macA	30	10	BAB1_1685	12 Λ	Ϊ	1 Ψ	efflux transporter, RND family, MFP subunit
210	9	BR0605	feuQ	30	10	BAB1_0629	1 Ψ	Ϊ	1 Ψ	Sensor protein phoQ
248	7	BRA0065	virB	30	10	BAB2_0064	Ϊ	1 Ψ	1 Ψ	P-type DNA transfer protein VirB5
268	9	BRA0299	narG	30	3	BAB2_0904	1 Ψ	Ϊ	1 Ψ	nitrate reductase, alpha subunit
122	9	BruAb2_0827	Catalase	34		BruAb2_0827	1 Ψ	Ϊ	1 Ψ	KatA, catalase
135	6	BAB1_0108	cgs	35		BruAb1_0108	1 Ψ	Ϊ	1 Ψ	cyclic beta 1–2 glucan synthetase
247	7	BR1241	K19	36		BruAb1_1246	Ϊ	1 Ins.	1 Ψ 1Λ	hypothetical protein
222	9	BR1296	K41	36		BruAb1_1350	1 Ψ	1 Ψ	Ϊ	hypothetical protein
248	7	BruAb2_0065	VirB5	40		BruAb2_0065	Ϊ	1 Ψ	1 Ψ	type IV secretion system protein VirB5

Pr. = Priority and slno = serial numbers as in Table S4. Ψ = bp Difference, Ref = Reference, ATT = Combined attenuation score, Λ  = Insertion\deletion, Ϊ = Identical.

Besides the comparisons of the sequences of the ORFs of the 236 virulence factors, the intergenic regions upstream of these genes (and all other ORFs in the genome) were also used to compare the attenuated and virulent strains. None of the differences at the intergenic regions were found to be consistent between S19 and both the virulent strains (data not shown).

### Conclusions

The *Brucella abortus* strain S19 is a spontaneously attenuated strain, which has been used as a vaccine strain in vaccination of cattle against brucellosis for six decades [4], [5]. Although it has been studied extensively, the physiological and molecular mechanisms causing the attenuation are not known [20]. The classical studies that evaluated the attenuation of S19 were the discovery of partial deletion and loss of function of two proteins ery operon (eryCD), and their characterization and mutational analysis [16]–[22]. At least in murine models, the deletion of these two genes was not enough to cause attenuation, suggesting that S19 has lost some additional and essential, yet unknown, mechanism of virulence in mice.

We have determined the complete genome sequence of S19 and conducted a comprehensive comparative analysis using the whole genome sequence of two virulent strains, 9–941 and 2308 and the newly sequenced attenuated strain, S19. Our comparative analyses agreed with previous studies to reveal >99% homology among the genomes sequences [10]. The differences in the method of gene prediction used in three different genomes has been corrected and shown on the PATRIC website (http://patric.vbi.vt.edu/). We conducted pairwise and reciprocal gene-to-genome comparisons to identify all of the 263 non-identical differences between S19 and the two virulent genomes, out of which only 45 ORFs or “OCDs” were consistently different between the attenuated S19 strain and both the virulent strains, 9–941 and 2308.

Among the 45 OCDs, only four ORFs had more than 60 nt difference between S19 and the virulent strains with no difference observed within the virulent strains (priority “0” in Table 7). The results revealed one additional ORF encoding protein involved in erythritol uptake (eryF), while confirming the previous findings on the 703 nt deletion in eryCD. We also identified an outer membrane protein with 768 aa deletion in S19. Although this difference is consistent when compared to the virulent strains of *B. abortus*, the virulent strain of *B. suis* also contain this deletion, making it a less probable candidate for attenuation. However, its role within *B. abortus* needs testing.

Besides the four major differences, we identified a prioritized list of 24 OCDs with minor but consistent differences between S19 and the two virulent strains, which included eight OCDs with frameshifts (priority 1 and 2), 12 OCDs with aa changes that cause a change in the net charge of the protein (priority 3) and nine OCDs with aa changes without change in the net charge (priority 4). Some of the intriguing differences with possible relevance to attenuation include four proteins involved in lipid transport and metabolism, two proteins involved in transcription, two transporter proteins, two outer membrane proteins, and several hypothetical proteins. We believe the characterization of these few proteins using mutation and host response studies will yield an account of the attenuation of S19.

## Materials and Methods

### Strain Information, Extraction and Characterization of The Genomic DNA

*B. abortus* S19 was obtained from the National Animal Disease Center collection. It was originally isolated from the milk of American Jersey Cattle by Dr. John Buck in 1923 [4], [5]. Total genomic DNA was extracted and purified by the modification of a previously described method [12]. An aliquot of the DNA was subjected for analysis using the Bioanalyzer (Agilent technologies) and was confirmed for no degradation of the DNA. An aliquot of 10ug of DNA was used for the sequencing via pyrosequencing (see below), and the remaining stock was maintained for further sequencing and completion of the gaps.

### Genome Sequencing, Whole Genome Assembly

The first round of high-throughput sequencing was performed via pyrosequencing [14]. A total of two, four-hour runs were performed to generate a total of ∼800 thousand sequences with an average length of about 100 bases, resulting in more than 20X coverage of the whole genome of the strain. The quality filtered reads were then assembled into contigs using the Newbler assembler (http://www.454.com/). A total of 701 contigs with at least two contributing fragments were formed, of which 172 contigs had sequence lengths ranging from 0.5 to 123 kb, with an average of 18.8kb.

The 172 contigs were aligned to the whole genome sequence of the *B. abortus* 9–941 [12] to identify the putative gaps to be sequenced in the whole genome of the *B. abortus* strain S19. Primers were designed and the genomic DNA from the *B. abortus* strain S19 was used as a template in PCR to amplify the segments that needed to be sequenced. The purified PCR amplicons were used as templates in sequencing. The newly generated sequences, together with the contigs, were used to determine the whole genome sequence.

### Gene Prediction and Annotation

Putative protein-encoding genes of the S19 genome were identified with Glimmer [43]. Genes consisting of fewer than 33 aa were eliminated, and those containing overlaps were manually evaluated. Start sites of each predicted gene were tuned by TiCO [44]. Sequences from the intergenic regions were compared to non-redundant protein databases to identify genes missed by the Glimmer prediction. tRNAs were identified with tRNAscan-SE [45], while ribosomal RNAs were identified by comparing the genome sequence to the rRNA database [46]. The whole genome sequence of *B. abortus* S19 is deposited in GenBank (accession # CP000887 and CP000888). The whole genome sequence and existing annotations were also submitted for additional curation (gene prediction and protein annotation) using the genome annotation pipeline of the PathoSystems Resource Integration Center (PATRIC) [47] and are made available at http://patric.vbi.vt.edu/.

### Comparative Genomics

Whole genome sequences and the predicted gene sets of *B. abortus* 2308 and 9–941 were downloaded from NCBI RefSeq [48]. SNPs between S19 and 9–941 genomes, as well as between S19 and 2308 genomes, were identified by mapping all of the 800,000 reads generated by the 454 machine to the reference genomes (9–941 and 2308, respectively) using the 454 whole genome mapping software which includes a high-confidence SNP identification module–MutationDetector (http://www.454.com).

To identify potential genes that differ between the attenuated strain S19 and other two *B. abortus* virulent strains, 2308 and 9–941, pair-wise and reciprocal comparisons were performed by aligning the predicted genes of one strain to the whole genome sequence of the second strain and vice versa. The genomes were automatically compared for missed gene calls, indels, frameshifts, and other sequence variants by a program called GenVar [49]. The improved annotations of the ORFs were performed by PATRIC and are made available at http://patric.vbi.vt.edu/.

## Supporting Information

Table S1 Functional assignment of the ORFs of *Brucella abortus* Strain S19 using BLASTP searches against the protein datasets
(0.54 MB XLS)

Table S2 Single Nucleotide Polymorphisms (SNPs) identified between S19 genome and 9–941 genome
(0.20 MB XLS)

Table S3 Pair-wise and reciprocal comparisons between the genes and the genomes of the strains S19, 9–941 and 2308
(1.29 MB XLS)

Table S4 List of genes identified to be different between attenuated strain S19 genome and the virulent strains, 9–941 and 2308
(0.09 MB XLS)
